# Exploring Dietary Intake in Adults with Type 2 Diabetes Using GLP-1 Receptor Agonists: A Cross-Sectional Analysis

**DOI:** 10.3390/nu17213318

**Published:** 2025-10-22

**Authors:** Valentina Ponzo, Marilena Vitale, Simona Bo, Fabio Broglio, Ilaria Goitre, Iolanda Cioffi

**Affiliations:** 1 Department of Medical Science, University of Torino, c.so Dogliotti 14, 10126 Torino, Italy; simona.bo@unito.it (S.B.); fabio.broglio@unito.it (F.B.); ilaria.goitre@unito.it (I.G.); 2 Nutrition, Diabetes and Metabolism Research Unit, Department of Clinical Medicine and Surgery, Federico II University of Naples, Via S. Pansini 5, 80131 Naples, Italy; marilena.vitale@unina.it; 3 Division of Human Nutrition, Department of Food, Environmental and Nutritional Sciences—DeFENS, University of Milan, Via G. Celoria 2, 20133 Milan, Italy; iolanda.cioffi@unimi.it

**Keywords:** GLP-1 receptor agonists, Type 2 diabetes, dietary intake, Mediterranean diet, nutritional adequacy, Pharmacotherapy, diet quality

## Abstract

**Background:** Glucagon-like peptide-1 receptor agonists (GLP-1RAs) are increasingly used in type 2 diabetes (T2D) management for their glycemic and weight benefits. However, their appetite-suppressing effects may influence dietary intake and nutrient adequacy, yet real-world evidence is scarce. **Objective:** To evaluate dietary intake and adherence to the Mediterranean diet in adults with T2D treated with GLP-1RAs compared to those receiving other oral hypoglycemic agents. **Methods:** In this cross-sectional study, 103 adults with T2D (mean age 66 ± 8 years; 65% male) attending a diabetes clinic in Turin, Italy, were enrolled between February and June 2025. Dietary habits were assessed using a validated food frequency questionnaire, and adherence to the Mediterranean diet was evaluated via the Mediterranean Diet Score (MDS). Anthropometric, biochemical, and lifestyle data were collected. **Results:** Fifty-two participants (50.5%) were treated with GLP-1RAs (semaglutide 55.8%, dulaglutide 40.4%). No significant differences in energy intake, macronutrient distribution, or MDS were observed between groups. Overall, diets were characterized by low carbohydrate intake (~44% of energy), inadequate fiber (≈11 g/1000 kcal), and high fat intake (≈39–40% of energy), with saturated fat below 10%. None of the GLP-1RA users met fiber recommendations. Subgroup analysis by treatment duration (<1 year, 1–2 years, >2 years) revealed no significant differences in dietary patterns. **Conclusions:** Patients with T2D, regardless of pharmacological treatment, exhibited poor adherence to dietary guidelines. These findings highlight the need for structured nutritional counseling alongside GLP-1RA therapy to optimize metabolic outcomes and prevent nutritional deficiencies.

## 1. Introduction

Type 2 diabetes (T2D) represents a major global health challenge and remains one of the leading causes of death and disability worldwide [1]. A recent global forecasting study estimated that over 500 million individuals were living with diabetes in 2021, with projections indicating a continued rise by 2050, particularly among aging populations and in low- and middle-income countries [2]. While lifestyle modification continues to be the cornerstone of T2D prevention and management, pharmacological therapy is often required to achieve and sustain adequate glycemic control and reduce the risk of long-term complications [3].

Among the various pharmacological options, glucagon-like peptide-1 receptor agonists (GLP-1 RAs) have emerged as a highly effective class of drugs due to their combined ability to improve glycemic control and promote clinically significant weight loss [4,5]. The benefits in weight reduction are primarily linked to delayed gastric emptying, appetite suppression, and increased satiety, which have been shown to lead to a reduction in energy intake of approximately 16% to 40% in individuals with overweight or obesity [6]. However, the appetite-suppressing effects of GLP-1 RAs may also lead to unintended nutritional consequences [7], especially when used in the absence of appropriate dietary counseling. With reduced caloric intake and reduced appetite, it is possible that individuals taking GLP-1 or GIP/GLP-1 RAs may reduce the overall intake of micronutrients [6]. A recent study in individuals with obesity treated with GLP-1 RAs reported inadequate intake of several essential nutrients, including calcium, iron, magnesium, and vitamins A, C, and D [8]. Moreover, their diets were often characterized by insufficient protein and fiber consumption alongside a high intake of total and saturated fats. These dietary patterns may be driven both by medication-induced gastrointestinal side effects and by a general lack of structured nutritional guidance [8]. This is particularly concerning in light of the increasing adoption of GLP-1 receptor agonists as long-term, and potentially lifelong, treatment strategies in the management of T2D [9].

Despite the widespread and growing use of GLP-1 RAs, particularly in diabetic patients with overweight or obesity [10], little is known about how these medications influence habitual dietary intake in real-world settings. Furthermore, it remains unclear whether the duration of treatment with GLP-1 RAs is associated with progressive changes in food intake or nutrient adequacy over time. These questions are critical, as unrecognized nutrient deficiencies could negatively impact metabolic health, body composition, and long-term adherence to treatment [11].

Therefore, the aim of this observational study was to assess dietary intake and adherence to the nutritional recommendations in patients with T2D treated with GLP-1 RAs, comparing them with those receiving other oral hypoglycemic agents, and eventually, to explore whether macronutrient intake varies according to the duration of GLP-1 RAs therapy. To the best of our knowledge, this is the first real-world investigation study, assessing the overall dietary intake and adherence to the Mediterranean diet in a cohort of Italian diabetic patients treated with GLP-1RAs, which aimed to explore their dietary habits in order to identify potential drawbacks due to treatment.

## 2. Materials and Methods

### 2.1. Study Population

This is a cross-sectional study enrolling patients with T2D who attended visits at the Diabetes and Metabolic Diseases Clinic, “Città della Salute e della Scienza” Hospital of Turin in Italy from February to June 2025. Patients were enrolled if they met the following inclusion criteria: age between 40 and 75 years, diagnosis of T2D, and treatment with either GLP-1 receptor agonists or oral hypoglycemic agents initiated at least 1 month prior to the enrollment. Patients were excluded if they were receiving insulin therapy, either alone or in combination with other agents, or if they had any medical conditions that could affect food intake. Additionally, individuals who were pregnant or lactating, as well as those unable to provide informed consent, were excluded from the study. GLP-1 receptor agonists were prescribed within the therapeutic ranges commonly used in clinical practice for patients with T2D, with the following ranges: liraglutide 1.2–1.8 mg/day, dulaglutide 0.75–1.5 mg/week, and semaglutide 0.25–1 mg/week.

### 2.2. Data Collection

During the follow-up visit at the center, all participants provided socio-demographic information and underwent clinical and dietary assessments, along with anthropometric measurements.

Socio-demographic data included participants’ age, sex, educational level, economic status, family composition, and smoking habits.

Dietary habits were collected using a medium-length food frequency questionnaire (FFQ), which has been validated for use in the Italian population [12]. This semi-quantitative tool evaluated habitual consumption patterns over the preceding year, focusing on 36 food items commonly consumed in Italy. Participants reported both the frequency and approximate quantity of intake for each item, selecting portion sizes from predefined options illustrated with reference images or standard household measures (e.g., cups, spoons, slices). To improve accuracy in estimating portion sizes, standardized food photographs were provided. The food items were organized into categories such as beverages (including coffee, alcoholic drinks, and soft drinks), dairy products, meat, fish, and eggs, cereals, fruits and vegetables (including legumes), fats and dressings, and miscellaneous items like sweets, fried foods, and fast food. Total energy (measured in kilocalories) and macronutrient intake (expressed both in grams and percentage of total calories) were calculated using WinFood® Pro 3.37.x software (Medimatica, Colonnella, Teramo, Italy, 2023).

The MDS (Mediterranean Diet Score) was calculated to assess participants’ adherence to the Mediterranean diet, as it is a validated index in Italian and European populations with T2D and specifically adapted to the Mediterranean dietary context. The score was constructed following the method originally proposed by Trichopoulou et al. [13], assigning one point for each typical Mediterranean diet component. Specifically, nine food groups were considered: high consumption of vegetables, fruits and nuts, legumes, cereals, fish, and a high ratio of monounsaturated to saturated fats; low consumption of meat and meat products; and moderate alcohol intake. For each component, participants with consumption above the median (or below, for “unhealthy” components) were assigned one point. The total score ranges from 0 to 9 with higher values indicating greater adherence [13].

Physical activity levels were assessed using the International Physical Activity Questionnaire (IPAQ) short form [14]. This validated tool measures the frequency and duration of walking, moderate, and vigorous physical activities performed during the previous seven days, allowing classification of participants into different activity levels (low, moderate, high).

Body weight was recorded with participants wearing light clothing and no footwear, using a mechanical column scale (SECA model 711, Hamburg, Germany) with a precision of 0.1 kg. Height was measured to the nearest 0.1 cm using a SECA 220 stadiometer (Hamburg, Germany). Waist and neck circumferences were taken with a plastic measuring tape, positioned at the umbilicus level [15] and on the cricoid cartilage [16], respectively.

Eventually, the presence of other co-morbidities and/or diabetes complications, disease duration as well as biochemical parameters including HbA1c, fasting blood glucose and lipid profile were extracted from patients’ medical records.

### 2.3. Statistical Analysis

Data analyses were performed using IBM SPSS (version 28, Chicago, IL, USA). Continuous variables were reported as means mean ± standard deviation (SD), unless otherwise specified, whilst categorical variables were expressed as frequencies and percentages. The Kolmogorov–Smirnov test and the Shapiro–Wilk test were used to examine whether variables were normally distributed. Unpaired *t*-test or Whitney U test was used for continuous and non-continuous variables to determine differences in anthropometric, biochemical and nutritional variables between patients taking GLP-1RAs and those undertaking other medications. The chi-square or Fisher’s exact tests were applied for categorical variables. The analysis of variance (ANOVA) or the Kruskal-Walli’s test were used to compare variables between more than two groups, and post hoc comparisons were made where appropriate. Finally, Spearman’s linear correlation was applied to evaluate correlations between adherence to Mediterranean Diet, dietary intake, age and anthropometric variables. Significance was defined as *p* < 0.05.

Although no a priori sample size calculation was performed, a post hoc power analysis was conducted using protein intake (expressed as percentage of total energy) as the benchmark variable, given its role on nutritional status. Using 15% of total energy from protein as the reference value recommended by dietary guidelines, the analysis indicated a statistical power of 81% (α = 0.05, two-tailed *t*-test), supporting the adequacy of the sample size for this outcome.

### 2.4. Ethical Considerations

All participants provided written informed consent prior to their inclusion in this study. The research protocol was reviewed and approved by the local Ethics Committee of Città della Salute e della Scienza di Torino hospital (protocol number CS/662 of 19 October 2015). All procedures were conducted in accordance with the ethical standards of the Declaration of Helsinki.

## 3. Results

A convenient sample of 103 patients with T2D (67 males and 36 females) were recruited for this cross-sectional analysis. Participant flow, from initial screening to exclusions and final analyzed sample, is reported in Supplementary Figure S1 Patients showed a mean age of 66 ± 8 years old and an average BMI of 28.1± 5.52 kg/m^2^ whose 40% of them were overweight and 30% were living with obesity. A full description of the whole sample characteristics, including socio-demographic information, is reported in Table 1.

Regarding T2D medications, 50% of patients were GLP-1 RAs users (semaglutide 55.8%, dulaglutide 40.4%, exenatide 3.8%) and the other half took other medications, mostly metformin (90.2%) and sodium-glucose cotransporter-2 (SGLT2) inhibitors (31.4%) either alone or in combination. Patients under GLP-1RAs showed an increased weight (*p* = 0.001), BMI (*p* = 0.001) and waist circumference (*p* = 0.008) compared with those under other medications, while no differences were observed for age, height, disease duration, prevalence of comorbidities and IPAQ classification between the two groups. Conversely, among the biochemical parameters, serum values of total cholesterol (*p* = 0.02) and LDL-cholesterol (*p* = 0.04) were lower for GLP-1RA users compared to patients taking other medications. HbA1c levels were also slightly lower in the GLP-1RA group, although the difference did not reach statistical significance (*p* = 0.055).

### 3.1. Comparison of Nutrient Intake and Adherence to the Mediterranean Diet Score Between Patients Taking GLP-1RAs Versus Those Under Other Medications

Energy and macronutrients intake retrieved from the semi-quantitative FFQ were calculated in both absolute and percentage values for both GLP-1RAs and other medications users and shown in Table 2. Results were similar in both groups, except for monounsaturated fatty acids (MUFA) resulting higher for GLP-1 users (*p* = 0.03) and characterized by low intakes of carbohydrates (44% of energy intake -EI-), total (~16 g) and adjusted dietary fibers per 1000 kcal/day (~11 g/1000 kcal) as well as by a high intake of fats (39–40% of EI), with saturated fatty acids (SFAs) below 10% of EI. The macronutrient distribution observed in both groups was not in line with the recommendations proposed by the European Association for the Study of Diabetes (EASD) dietary guidelines, as shown in Table 2. Similar data were observed in both absolute and percentage data when the analysis was separated by sex (Table S1).

**Figure 1:**
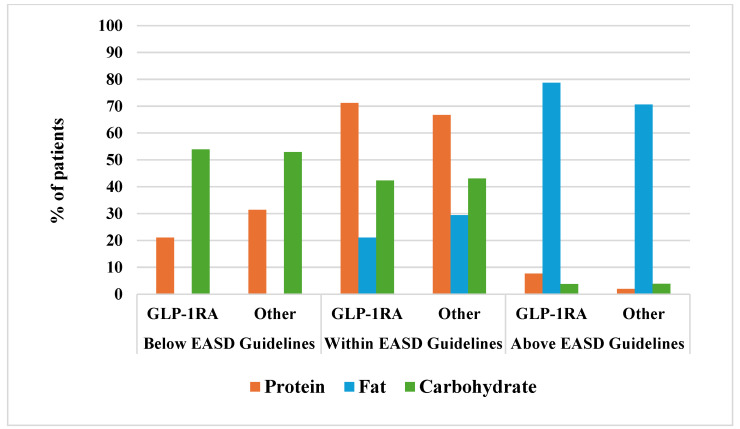
Percentage of patients with T2D meeting or not EASD Guidelines for each macronutrient.

In Figure 1, the percentage of patients meeting or not the dietary guidelines are presented, highlighting that the intake of carbohydrate was reduced in over 50% of patients, whereas fats were elevated in about 70% of them, unrelated to drugs treatment. Also, we found that none of the patients in GLP-1RAs group, but just one in the other group met fiber recommendations, whereas high intake of soluble sugars was shown in over 50% of patients (>10% of EI) (Figure S2).

**Figure 2:**
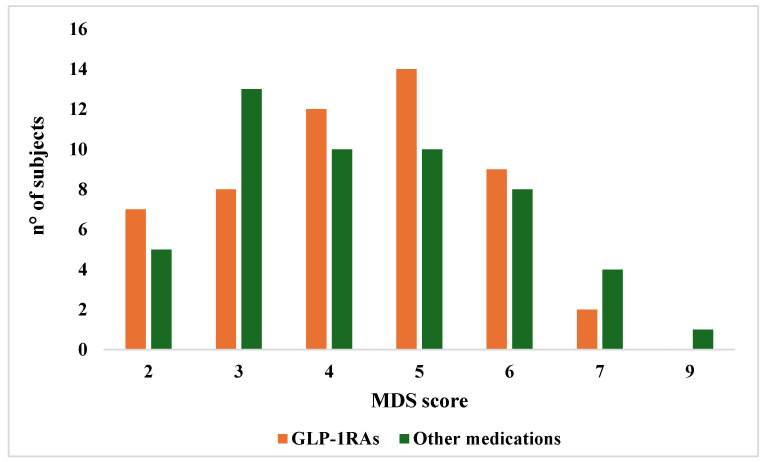
Mediterranean Diet Score distribution according to treatment.

Eventually, the adherence to the Mediterranean diet assessed by the MDS index did not show any significant differences between the GLP-1RAs and other medications group (MDS mean ± SD: 4.31 ± 1.39 vs. 4.39 ± 1.60; *p* = 0.78; median [range]: 4 [2,3,4,5,6,7] vs. 4 [2,3,4,5,6,7,8,9]; *p* = 0.82). Score distributions for both groups are presented in Figure 2.

### 3.2. Correlations Between MDS, Nutrient Intake, Age and Anthropometric Variables

Sperman’s linear correlations showed that the MDS was directly correlated to age (r = 0.220, *p* = 0.025), soluble sugar (r = 0.201, *p* = 0.039), total fiber intake (r = 0.416, *p* < 0.001) and fiber adjusted for 1000 kcal (r = 0.480, *p* < 0.001) but inversely associated with protein (r = −0.197, *p* = 0.046) and cholesterol intake (r = 0.252, *p* = 0.010) in the whole sample. Repeating the analysis for each group, the same results were found in the other medications, apart from age, while MDS was positively correlated to age (r = 0.292, *p* = 0.036), total fiber (r = 0.317, *p* = 0.022) and fiber adjusted for 1000 kcal (r = 0.368, *p* = 0.007) in GLP-1RAs users.

### 3.3. Comparison of Nutrient Intake in GLP-1RAs Users According to Treatment Duration

To evaluate how dietary habits might be affected according to treatment duration, since patients started taking GLP-1RAs at different time points, a subgroups analysis was performed by splitting the whole sample into 3 subgroups as follows: patients taking medications for less than 1 year (<1 y), those taking mediations between 1 and 2 years (1–2 y) and the last group using GLP-1RAs for over 2 years (>2 y). Data on both energy and macronutrients are shown in Table 3. Overall, we did not find any significant differences in energy and macronutrient intake among the 3 subgroups.

**Figure 3:**
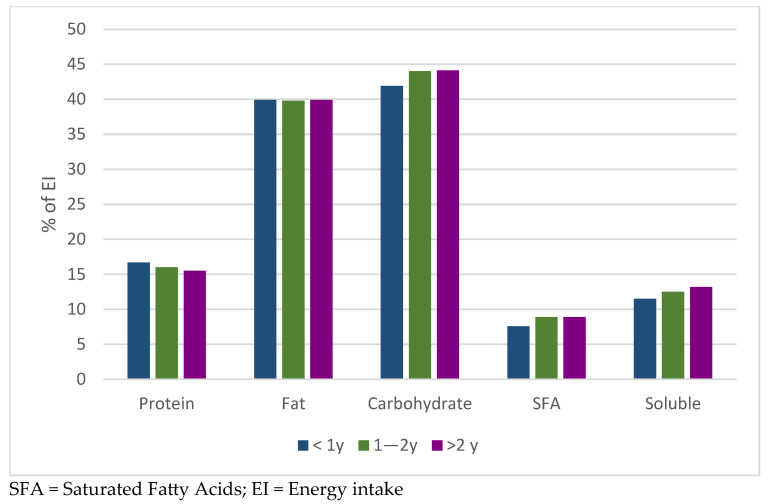
Macronutrient distribution according to GLP-1RAs duration.

Percentage values for macronutrients, SFA and soluble sugars were presented in Figure 3, where it seemed that those taking medications for less than 1 year showed a slightly higher intake of protein and lower intake of carbohydrates than the other two groups, without being statistically significant.

### 3.4. Comparison of Weekly Frequency Consumption of the Main Food Groups in GLP-1RAs Users According to Treatment Duration

**Figure 4:**
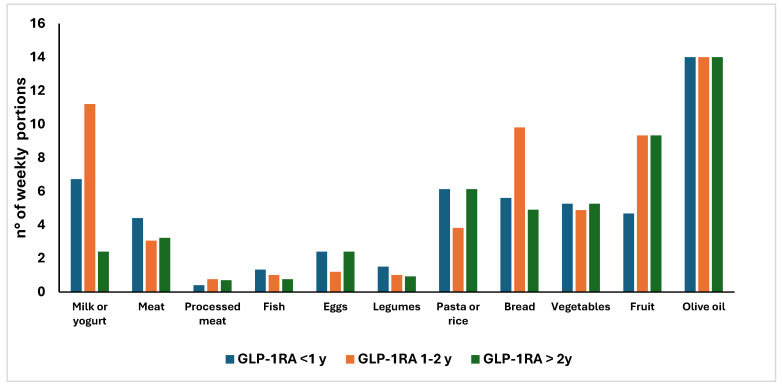
Weekly consumption of food groups according to GLP-1RAs duration.

Finally, we looked into the weekly consumption of the main food or food groups retrieved from the FFQ according to the 3 subgroups of GLP-1RAs users, considering as reference portion for each food group the one proposed by the fifth version of LARN [17]. As reported in Figure 4, no significant differences were found in the number of consumed portions among patients, due to their huge variability. However, the median weekly consumption of different foods such as fish or legumes as well as of vegetables and fruit did not reach the dietary recommendations. Also, some patients did not eat some food at all.

## 4. Discussion

In this cross-sectional study evaluating dietary intake among patients with T2D either treated or not with GLP-1RAs, we observed generally poor diet quality across both groups, with most individuals failing to meet established nutritional guidelines and reporting low adherence to the Mediterranean diet, unrelated to therapy. In addition, total energy intake, macronutrient composition or adherence to the Mediterranean diet were similar between patients treated with GLP-1RAs and those receiving other oral hypoglycemic agents.

Connecting these findings to current nutritional recommendations, we further analyzed dietary intake regardless of treatment type. The data revealed an overall excessive intake of total and saturated fats, along with low carbohydrate consumption and inadequate fiber intake—the latter unmet by all GLP-1RA users and by only one patient from the control group, according to recommendations. Moreover, adherence to the Mediterranean diet was also low and did not differ significantly according to pharmacological treatment. These results are consistent with previous studies in patients with T2D, which have similarly reported persistently low adherence to dietary guidelines [18,19], particularly regarding fiber and fat intake [20], as well as poor adherence to the Mediterranean dietary pattern [21,22].

There is limited literature specifically assessing dietary intakes in individuals with T2D undergoing treatment with GLP-1RAs. A small Japanese study involving 34 patients with T2D and obesity treated with semaglutide demonstrated significant improvements in eating behaviors, including reductions in hunger, emotional eating, and irregular eating patterns during treatment [23]. A study of Jensen SBK et al. [24] involving 215 individuals with obesity and treated with liraglutide showed a decreased consumption of high-fat foods and a significant reduction in appetite compared to the placebo group.

Our findings are partially consistent with those reported by Johnson et al. [8], which highlighted that individuals with obesity undergoing treatment with GLP-1 receptor agonists (GLP-1RAs) often fail to meet the Dietary Reference Intakes (DRIs) for several essential nutrients. In our cohort, we observed a similarly unbalanced dietary profile among patients treated with GLP-1RAs. Specifically, their dietary intake was marked by a notable deficiency in carbohydrates and dietary fiber, coupled with an excessive consumption of total fats and saturated fatty acids (SFAs). Interestingly, protein intake remained within the recommended range, suggesting that while some macronutrient targets were met, others were significantly misaligned with nutritional guidelines. These findings underscore the need for tailored dietary counseling in patients receiving GLP-1RA therapy to mitigate potential nutritional imbalances and optimize overall health outcomes.

In patients with type 2 diabetes and obesity, GLP-1RAs therapy has been associated with appetite suppression and alterations in food preferences, often resulting in reduced consumption of energy-dense, high-fat foods and decreased food cravings [6,25,26,27,28,29]. Therefore, we would have expected to observe significant differences in dietary intake between patients treated with GLP-1RAs and those receiving other hypoglycemic therapies. However, no significant differences emerged between the treatment groups in terms of energy or macronutrient intake, nor in adherence to dietary recommendations. One possible explanation could be the relatively low doses of GLP-1RAs prescribed in patients with diabetes, which are lower than those used in obesity-focused regimens [30]. This may have resulted in modest effects on appetite regulation and food preferences, potentially limiting dietary changes. It is also important to acknowledge that patients in our study treated with GLP-1RAs had significantly higher BMI and waist circumference, which is expected since clinical guidelines recommend GLP-1RAs use in patients with T2D with obesity or increased cardiometabolic risk [31]. Moreover, information about pre-existing dietary habits has not been systematically collected, representing a potential bias to deal with. Therefore, the lack of significant dietary differences observed between the groups in our study should be interpreted considering those disparities.

Another possible explanation is that individuals with higher BMI might be more susceptible to underreporting dietary intake, particularly in self-reported assessments [32]. The relatively low reported energy intake compared to expected requirements may reflect the known underreporting bias of self-reported dietary questionnaires, particularly among individuals with higher BMI, but could also be related to adherence to prescribed hypocaloric regimens in the context of diabetes and weight management. This dual possibility makes it difficult to disentangle true intake from reporting bias and should be taken into account when interpreting our findings. The potential underestimation could have led to a convergence in reported dietary intake across groups, thereby hiding potential pharmacological effects on dietary intake. Additionally, individuals with obesity may have already undergone dietary counseling or implemented hypocaloric diets prior to initiating GLP-1RAs therapy, further attenuating observable differences.

Interestingly, our subgroup analysis based on duration of GLP-1RAs use (<1 year, 1–2 years, >2 years) did not reveal significant changes in dietary intake over time. This was somewhat unexpected given that gastrointestinal side effects typically peak during the first months of GLP-1RA therapy and tend to stabilize thereafter, often leading to an early reduction in food intake [33]. However, the small subgroup sizes limited the statistical power to detect differences, preventing us from ruling out the possibility of dietary modifications over time. This warrants further investigation in larger longitudinal studies.

Still, as previously mentioned, the GLP-1RAs dose might have played a role in blunting the pharmacodynamic effects on appetite and dietary intake, potentially explaining why no differences were observed on food intake, even shortly, after treatment initiation.

Therefore, regardless of the type or duration of pharmacological treatment, patients with T2D in our study exhibited consistently inadequate dietary patterns, underscoring the need for individualized nutritional interventions. Prior studies have shown that only a minority of patients with T2D receive tailored dietary counseling, while the majority are given general dietary advice by diabetologists [18]. Such a gap in care may be especially critical in the context of GLP-1RAs therapy, given their known effects on appetite regulation and food preferences, which require appropriate nutritional oversight [6]. Concerns have also been raised about subclinical micronutrient deficiencies in patients treated with GLP-1RAs. In a recent real-world, retrospective observational study, nutritional deficiencies were identified in 12.7% of T2D patients within six months of GLP-1RA initiation, and in 22.4% within twelve months, vitamin D deficiency being the most frequently observed [34]. The lack of routine nutritional screening or follow-ups in clinical practice may therefore represent a missed opportunity for timely intervention and prevention of potential complications.

### 4.1. Clinical Implications

Taken together, our results highlight an excessive intake of total and saturated fats and an inadequate fiber consumption, irrespective of medication type, suggesting that concurrent nutritional interventions remain essential. From a clinical perspective, the present findings reinforce the need for structured and individualized nutritional counseling to be systematically included into diabetes care management, even in patients treated with GLP-1RAs—both at therapy initiation and during follow-up, to monitor and address potential nutritional imbalances and optimize clinical outcomes.

### 4.2. Limitations

Several drawbacks must be acknowledged. An intrinsic limitation of this study is its descriptive and cross-sectional design, which does not allow for inferences or conclusions regarding causal relationships between GLP-1RA treatment and dietary intake. Nevertheless, it provides valuable real-world evidence on dietary patterns in patients with T2D, helping to identify potential gaps that require further investigation.

Although the semi-quantitative FFQ used in this study is validated for the Italian population and provides an acceptable estimation of energy and macronutrient intake, it relies on participants’ recall and their ability to estimate portion sizes used to quantify intake, which may sometimes not precisely correspond to the food actually consumed. This limitation could introduce reporting bias—particularly among individuals with obesity [35]. However, results from the FFQ were carefully checked together with participants to ensure the accuracy and internal consistency of self-reported data. Such bias may nonetheless have attenuated true differences between groups, potentially leading to an underestimation of dietary variation. Furthermore, while this FFQ allows for reliable estimation of macronutrient intake, it is not sufficiently accurate to assess micronutrient adequacy, which represents a recognized limitation for self-reported dietary assessment tools. Given these potential drawbacks, it might be useful to incorporate more objective measures of dietary intake, such as nutritional biomarkers or digital food tracking, to enhance data reliability and minimize bias in collecting dietary intake data.

Another limitation is the small sample size that could have prevented us to detect potential differences between the two groups as well as among subgroups, even after adjusting data for confounding factors such as sex, race/ethnicity, age, or BMI. Furthermore, the lack of information about prior structured nutritional counseling and variations in both duration and dosage of GLP-1RA treatment could have, respectively, influenced eating habits and food intake response, potentially masking differences between groups; thus, residual confounding cannot be ruled out.

Moreover, as the sample was drawn from a single diabetes clinic, the results cannot be generalized to other populations or healthcare settings. Last, but not least, due to the nature of the study, we did not include systematic measurements of nutritional biomarkers (e.g., vitamins or micronutrient levels), which would have provided objective support for our findings. This limitation highlights the need for future prospective studies combining dietary questionnaires with biochemical markers of dietary intake to achieve a more accurate and multidimensional evaluation of nutritional status in this population.

Overall, these factors highlight that the findings should be interpreted as descriptive real-world evidence rather than causal relationships, and they emphasize the need for larger, prospective studies to further clarify these associations.

## 5. Conclusions

The use of GLP-1RAs in patients with T2D was associated with poor dietary intake and adherence to Mediterranean diet, in line with the results observed for patients taking other hypoglycemic therapies. Regardless of treatment, the overall dietary profile of patients did not meet the current recommendations, particularly concerning carbohydrate, fiber, and fat intake. These findings reinforce the necessity of integrating structured and personalized dietary support into diabetes care, also in case of starting GLP-1RAs therapy. However, further longitudinal studies are needed to assess the long-term nutritional implications of these treatments and the potential role of dietary counseling in optimizing clinical outcomes.

## Figures and Tables

**Table 1:** General characteristics of the included sample according to drugs treatment.

Variable	Overall Sample (*n* = 103)	GLP-1RAs (*n* = 52)	Other Drugs (*n* = 51)
Age, years	65.8 ± 7.9	65.0 ± 7.9	66.7 ± 7.8
Sex, *n* (%)			
Males	67 (65%)	35 (67%)	32 (63%)
Females	36 (35%)	17 (33%)	19 (37%)
Smoking status, *n* (%)			
Yes	12 (11.7%)	7 (13.5%)	5 (9.8%)
No	91 (88.3%)	45 (86.5%)	46 (90.2%)
Education, *n* (%)			
Primary school	9 (8.7%)	2 (3.8%)	7 (13.7%)
Secondary school	40 (38.8%)	21 (40.4%)	19 (37.2%)
High school	43 (41.7%)	22 (42.3%)	21 (41.2%)
University	11 (10.7%)	7 (13.5%)	4 (7.9%)
Marital status, *n* (%)			
Single	13 (12.6%)	7 (13.5%)	6 (11.8%)
Married	70 (68%)	38 (73%)	32 (62.8%)
Widow	11 (10.7)	2 (3.8%)	9 (17.6%)
Divorces	9 (8.7%)	5 (9.6%)	4 (7.8%)
Weight, kg	79.5 ± 18.3	85.2 ± 20.0	73.6 ± 14.0 **
Height, m	1.68 ± 0.09	1.68 ± 0.09	1.68 ± 0.08
BMI, kg/m^2^	28.1 ± 5.5	30.0 ± 5.9	26.1 ± 4.4 **
BMI categories, *n* (%)			
Normal	31 (30.1%)	9 (17.3%)	22 (43.1%)
Overweight	41 (39.8%)	20 (38.5%)	21 (41.2%)
Obesity	31 (30.1%)	23 (44.2%)	8 (15.7%)
Waist circumference, cm	103.1 ± 13.8	107 ± 13	99 ± 13 **
Disease duration, years	12.7 [0.4–47.5]	12.7 [0.4–47.5]	13.0 [0.7–45.5]
Comorbidities, *n* (%)			
Hypertension	53 (51.5%)	23 (44%)	30 (59%)
Dyslipidemia	55 (53.4%)	27 (52%)	28 (55%)
CKD	12 (11.7%)	5 (10%)	7 (14%)
MALSD	11 (10.7%)	6 (11%)	5 (10%)
Fasting blood glucose, mg/dL	144 ± 48	137 ± 49	151 ± 46
HbA1c, %	7.4 ± 1.7	7.0 ± 1.5	7.6 ± 1.8 *
Total cholesterol, mg/dL	159 ± 47	146 ± 47	169 ± 46 *
LDL cholesterol, mg/dL	85 ± 39	76 ± 42	93 ± 36 *
HDL cholesterol, mg/dL	49 ± 13	46 ± 11	51 ± 15
Triglycerides, mg/dL	135 ± 68	134 ± 52	136 ± 80
Physical activity (IPAQ), *n* (%)			
Sedentary	18 (17.5%)	12 (23.1%)	6 (11.8%)
Poor active	39 (37.9%)	16 (30.8%)	23 (45.1%)
Moderately active	37 (35.9%)	22 (42.3%)	15 (29.4%)
Active	8 (7.7%)	2 (3.8%)	6 (11.8%)
Very active	1 (1%)	-	1 (2%)

Data are expressed as mean ± standard, unless otherwise specified (median [min–max]). BMI categories: normal 18.5–24.9 kg/m^2^; overweight 25.0–29.9 kg/m^2^; obesity ≥ 30.0 kg/m^2^. BMI= body mass index; CKD = chronic kidney disease; GLP-1Ras = glucagon like peptide 1 receptor agonists; HbA1c = hemoglobin glycate; IPAQ = international physical activity questionnaire; MASLD = metabolic dysfunction-associated steatotic liver disease; y = year. ** *p* < 0.01; * *p* < 0.05.

**Table 2:** Daily energy and macronutrient intake in patients with T2D according to treatment and adherence to nutritional recommendations.

	EASD Guidelines	GLP-1 Ras (*n* = 52)	% of EI	Other (*n* = 51)	% of EI
Energy intake (EI), kcal/d		1514 ± 353		1568 ± 294	
Protein, g	10/15–20% °	60.5 ± 17.1	16%	60.1± 13.9	15%
Fat, g	20–35% EI	67.3 ± 20.5	40%	67.9 ± 18.2	39%
SFA, g	<10% EI	14.8 ± 6.14	9%	15.8 ± 7.52	9%
MUFA, g		29.2 ± 9.42	17% *	27.4 ± 7.97	16%
PUFA, g		7.77 ± 3.32	5%	7.87 ± 3.10	4%
n-6 PUFA, g		5.26 ± 1.85	3%	5.13 ± 1.47	3%
n-3 PUFA, g		0.77 ± 0.25	0.4%	0.74 ± 0.22	0.4%
Cholesterol, mg	-	209 ± 98.6		183 ± 71.3	
Carbohydrate, g	45–60% EI	176 ± 48.5	44%	183 ± 36.8	44%
Starch, g	-	115 ± 34.2		116 ± 34.5	
Soluble, g	<10% EI	51.1 ± 22.2	13%	49.0 ± 21.0	12%
Fiber/1000 kcal, g	16.7	11.0 ± 2.51		11.0 ± 2.81	
Total fibers, g	35	16.4 ± 4.54		16.4 ± 4.51	

° 10–20% if age < 65 y, 15–20% if age > 65 y. SFA: Saturated Fatty Acids; MUFA: Monounsaturated Fatty Acids; PUFA: Polyunsaturated Fatty Acids; EASD: European Association for the Study of Diabetes; EI: Energy intake. * *p* < 0.05.

**Table 3:** Daily energy and macronutrient intake according to GLP-1RAs duration.

	GLP-1RAs Treatment Duration		
	<1 y (*n* = 11)	1–2 y (*n* = 19)	>2 y (*n* = 22)
Energy intake, kcal/d	1511 ± 270	1488 ± 318	1538 ± 424
Protein, g	62.5± 11.3	59.7 ± 15.7	60.1 ± 20.9
Fat, g	67.5 ± 20.1	66.3 ± 20.5	68.1± 21.2
SFA, g	12.9 ± 5.73	15.1 ± 5.75	15.6 ± 6.72
MUFA, g	26.5 ± 6.52	29.1 ± 10.6	30.5 ± 9.68
PUFA, g	7.37 ± 3.62	7.91 ± 3.39	7.84 ± 3.31
n-6 PUFA, g	4.68 ± 1.31	5.12 ± 2.30	5.59 ± 1.57
n-3 PUFA, g	0.75 ± 0.16	0.75 ± 0.29	0.79 ± 0.24
Cholesterol, mg	223 ± 142	194 ± 61	212 ± 102
Carbohydrate, g	169 ± 43.4	173 ± 38.0	181 ± 59.4
Starch, g	120 ± 25.0	112 ± 29.4	115 ± 41.1
Soluble, g	49.1 ± 29.4	48.5 ± 13.9	53.9 ± 25.6
Fiber/1000 kcal, g	10.8 ± 3.0	11.1 ± 2.31	10.9 ± 2.52
Total fibers, g	16.4 ± 5.84	16.3± 3.50	16.4 ± 4.82

Data are expressed as mean ± standard deviation, unless otherwise specified. SFA: Saturated Fatty Acids; MUFA: Monounsaturated Fatty Acids; PUFA: Polyunsaturated Fatty Acids.

## Data Availability

The data presented in this study are available on request from the corresponding author due to privacy restriction.

## Supplementary Materials

The following supporting information can be downloaded at: https://www.mdpi.com/article/10.3390/nu17213318/s1, Table S1: Daily energy and macronutrient intake stratified by sex; Figure S1: participant flow diagram; Figure S2: Percentage of patients with T2D meeting or not EASD Guidelines for saturated fatty acids (SFA), soluble sugars and fiber intake.
