# Supplementary material for: Exploring Dietary Intake in Adults with Type 2 Diabetes Using GLP-1 Receptor Agonists: A Cross-Sectional Analysis
Source: Nutrients. 2025 Oct 22;17(21):3318. doi: 10.3390/nu17213318 (PMC12610383; doi:10.3390/nu17213318)
Supplement: Supplementary file 1 [file nutrients-17-03318-s001.zip › Table S1.pdf]

**Table S1.** Daily energy and macronutrient intake stratified by sex

| | Males | | Females | |
|---|---|---|---|---|
| | **GL1-RAs (n=35)** | **Other (n=32)** | **GLP1-RAs (n=17)** | **Other (n=19)** |
| **Energy intake, kcal/d** | 1540±318 | 1617±321 | 1463±423 | 1485±227 |
| **Protein, g** | 62.2±14.5 | 61.8±15.2 | 56.9±21.0 | 57.2±11.2 |
| **Fat, g** | 68.2±20.2 | 68.1±21.0 | 65.6±21.6 | 67.8±12.7 |
| SFA, g | 14.9±6.2 | 16.1±5.9 | 14.8±6.3 | 15.3±9.9 |
| MUFA, g | 29.3±9.7 | 28.0±8.4 | 28.9±9.0 | 26.5±7.3 |
| PUFA, g | 7.7±3.3 | 8.2±3.3 | 7.8±3.5 | 7.2±2.6 |
| n-6 PUFA, g | 5.3±2.1 | 5.4±1.5 | 5.2±1.3 | 4.6±1.4 |
| n-3 PUFA, g | 0.7±0.3 | 0.8±0.2 | 0.8±0.2 | 0.7±0.2 |
| **Cholesterol, mg** | 204.7±99.9 | 189.1±77.7 | 216.5±98.5 | 173.1±59.7 |
| **Carbohydrate, g** | 177.2±47.9 | 190.8±35.3 | 172.6±51.1 | 171.5±37.1 |
| Starch, g | 120.3±34.9 | 121.6±33.7 | 104.5±31.2 | 106.1±34.6 |
| Soluble sugars, g | 48.3±18.8 | 49.8±22.4 | 56.3±27.3 | 47.6±18.7 |
| **Fiber/1000 kcal, g** | 10.5±2.3 | 10.7±2.3 | 11.9±2.8 | 10.4±3.2 |
| **Total fibers, g** | 16.1±4.4 | 17.1±4.1 | 17.0±5.0 | 15.3±4.8 |

Data are expressed as mean ± standard deviation, unless otherwise specified. SFA: Saturated Fatty Acids; MUFA: Monounsaturated Fatty Acids; PUFA: Polyunsaturated Fatty Acids.
