# Supplementary figures and images for: Exploring Dietary Intake in Adults with Type 2 Diabetes Using GLP-1 Receptor Agonists: A Cross-Sectional Analysis
Source: Nutrients. 2025 Oct 22;17(21):3318. doi: 10.3390/nu17213318 (PMC12610383; doi:10.3390/nu17213318)

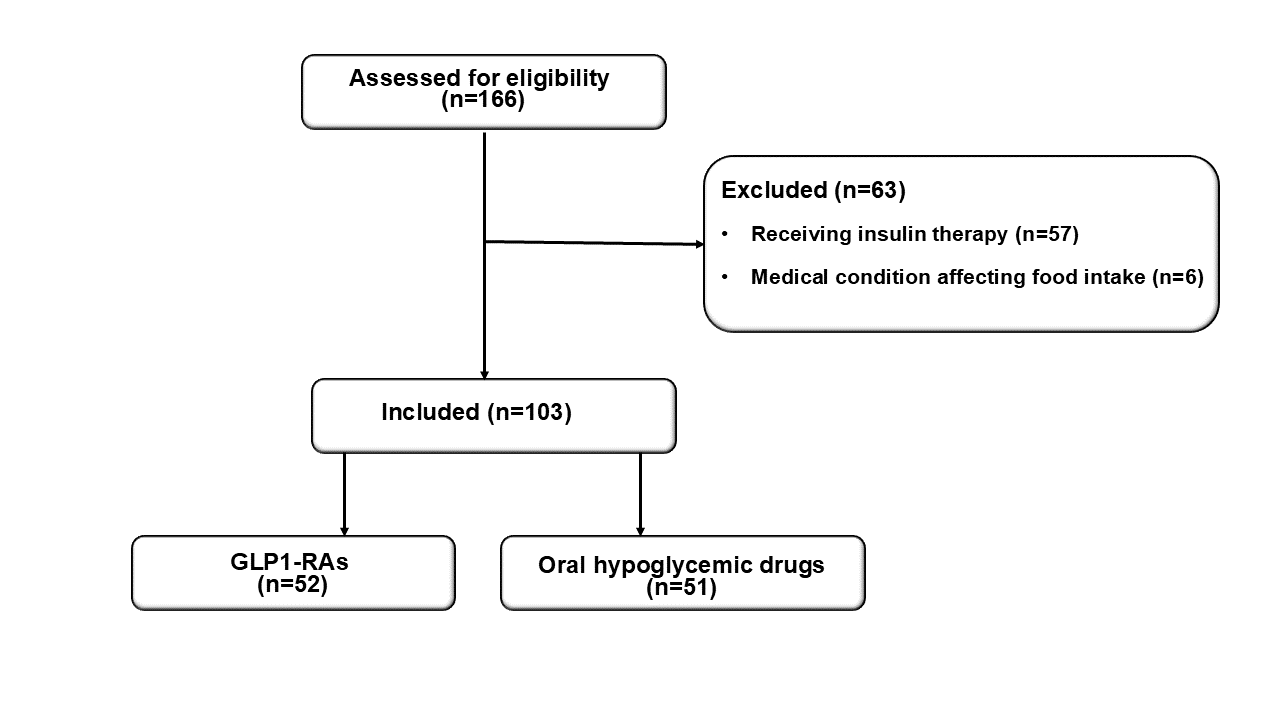

Supplement: Supplementary file 1 [file nutrients-17-03318-s001.zip › Figure S1.png]
